# Results of sub-analysis of a phase 2 study on trabectedin treatment for extraskeletal myxoid chondrosarcoma and mesenchymal chondrosarcoma

**DOI:** 10.1186/s12885-016-2511-y

**Published:** 2016-07-14

**Authors:** Hideo Morioka, Shunji Takahashi, Nobuhito Araki, Hideshi Sugiura, Takafumi Ueda, Mitsuru Takahashi, Tsukasa Yonemoto, Hiroaki Hiraga, Toru Hiruma, Toshiyuki Kunisada, Akihiko Matsumine, Michiro Susa, Robert Nakayama, Kazumasa Nishimoto, Kazutaka Kikuta, Keisuke Horiuchi, Akira Kawai

**Affiliations:** Department of Orthopaedic Surgery, Keio University School of Medicine, 35 Shinanomachi Shinjuku-ku, Tokyo, 160-8582 Japan; Department of Medical Oncology, Cancer Institute Hospital of Japanese Foundation for Cancer Research, Tokyo, Japan; Department of Orthopaedic Surgery, Osaka Medical Center for Cancer and Cardiovascular Diseases, Osaka, Japan; Department of Orthopaedic Surgery, Aichi Cancer Center Hospital, Aichi, Japan; Department of Orthopaedic Surgery, Osaka National Hospital, Osaka, Japan; Division of Orthopaedic Surgery, Shizuoka Cancer Center Hospital, Shizuoka, Japan; Division of Orthopaedic Surgery, Chiba Cancer Center, Chiba, Japan; Department of Orthopaedic Surgery, Hokkaido Cancer Center, Hokkaido, Japan; Department of Musculoskeletal Tumor Surgery, Kanagawa Cancer Center, Kanagawa, Japan; Department of Medical Materials for Musculoskeletal Reconstruction, Okayama University Graduate School of Medicine, Dentistry, and Pharmaceutical Sciences, Okayama, Japan; Department of Orthopedic Surgery, Mie University Graduate School of Medicine, Mie, Japan; Department of Musculoskeletal Oncology, Rare Cancer Center, National Cancer Center Hospital, Tokyo, Japan

**Keywords:** Extraskeletal myxoid chondrosarcoma, Mesenchymal chondrosarcoma, Trabectedin, Translocation-related sarcoma, Chemotherapy

## Abstract

**Background:**

Trabectedin is reported to be particularly effective against translocation-related sarcoma. Recently, a randomized phase 2 study in patients with translocation-related sarcomas unresponsive or intolerable to standard chemotherapy was conducted, which showed clinical benefit of trabectedin compared with best supportive care (BSC). Extraskeletal myxoid chondrosarcoma (EMCS) and Mesenchymal chondrosarcoma (MCS) are very rare malignant soft tissue sarcomas, and are associated with translocations resulting in fusion genes. In addition, the previous in vivo data showed that trabectedin affect tumor necrosis and reduction in vascularization in a xenograft model of a human high-grade chondrosarcoma. The aim of the present analysis was to clarify the efficacy of trabectedin for EMCS and MCS subjects in the randomized phase 2 study.

**Methods:**

Five subjects with EMCS and MCS received trabectedin treatment in the randomized phase 2 study. Three MCS subjects were allocated to the BSC group. Objective response and progression-free survival (PFS) were assessed according to the Response Evaluation Criteria in Solid Tumors (RECIST) version 1.1 by central radiology imaging review.

**Results:**

The median follow-up time of the randomized phase 2 study was 22.7 months, and one subject with MCS was still receiving trabectedin treatment at the final data cutoff. The median PFS was 12.5 months (95 % CI: 7.4–not reached) in the trabectedin group, while 1.0 months (95 % CI: 0.3–1.0 months) in MCS subjects of the BSC group. The six-month progression-free rate was 100 % in the trabectedin group. One subject with MCS showed partial response, and the others in the trabectedin group showed stable disease. Overall survival of EMCS and MCS subjects was 26.4 months (range, 10.4–26.4 months) in the trabectedin group. At the final data cutoff, two of five subjects were still alive.

**Conclusions:**

This sub-analysis shows that trabectedin is effective for patients with EMCS and MCS compared with BSC. The efficacy results were better than previously reported data of TRS. These facts suggest that trabectedin become an important choice of treatment for patients with advanced EMCS or MCS who failed or were intolerable to standard chemotherapy.

**Trial registration:**

The randomized phase 2 study is registered with the Japan Pharmaceutical Information Center, number JapicCTI-121850 (May 31, 2012).

## Background

Trabectedin is a marine-derived antitumor agent, initially isolated from the marine ascidian (*Ecteinascidia turbinata*) and currently produced synthetically. In 2007, trabectedin 1.5 mg/m^2^ as a single infusion lasting 24 h every 3 weeks was approved by the European Medicines Agency (EMA) for treatment of advanced soft tissue sarcomas in adults that had become unresponsive to anthracyclines and ifosfamide or when unsuited to receive these agents. It is currently in widespread use in Europe as a 2nd- or 3rd-line chemotherapeutic agent for the treatment of advanced soft tissue sarcoma. The antitumor mechanism of trabectedin is known to consist of selectively binding to the minor groove of DNA, and then interacting with the DNA excision and repair mechanism and a transcription inhibiting action, resulting in inhibition of cell division and induction of apoptosis and antiangiogenesis [1]. Trabectedin also interferes with the transcription of the oncogenic fusion proteins of translocation-related sarcomas (TRS) [2, 3]. The fusion proteins which were generated by chromosome translocation cause to change of phenotypic properties in cell to contribute to the tumorigenic pathway [4].

Recent clinical data showed specifically effectiveness of trabectedin against TRS; retrospective analysis of eight clinical studies reported encouraging disease control of trabectedin in TRS patients [5]. Based on this information, a randomized controlled phase 2 study of trabectedin 1.2 mg/m^2^ in patients with TRS who had failed or had been intolerable to standard chemotherapy was conducted in Japan. The overall median progression-free survival (PFS) of the 73 subjects with TRS in the randomized phase 2 study was 5.6 months (95 % CI: 4.1–7.5) in the trabectedin group and 0.9 months (95 % CI: 0.7–1.0) in the best supportive care (BSC) group, which showed that PFS was significantly prolonged in the trabectedin group in comparison with the BSC group [6].

Extraskeletal myxoid chondrosarcoma (EMCS) and Mesenchymal chondrosarcoma (MCS) are very rare malignant soft tissue sarcomas. Recent cytogenetic and molecular genetic studies of EMCS have found reciprocal translocations, typically t(9;22)(q22;q12.2), resulting in fusion of *EWSR1* to *NR4A3* [7, 8]. MCS is morphologically characterized by a biphasic pattern of undifferentiated round cells and islands of hyaline cartilage. Recently, the *HEY1-NCOA2* fusion gene has been also reported in MCS [9].

In addition, previous report shows that trabectedin affects tumor necrosis and reduction in vascularization in a xenograft model of a human high-grade chondrosarcoma [10], which suggests that trabectedin shows particularly high efficacy in EMCS and MCS because their cells are histopathologically similar to the human chondrosarcoma cell line.

In the present analysis, we assessed the efficacy of trabectedin especially against the very rare histological types EMCS and MCS in the above-described randomized phase 2 study.

## Methods

### Patients

As the subjects of this sub-analysis, we adopted two EMCS subjects and three MCS subjects who had been allocated to the trabectedin group and three MCS subjects who had been allocated to the BSC group in the randomized phase 2 study. The inclusion and exclusion criteria of the randomized phase 2 study have been previously described [6]. In brief, eligible patients were pathologically diagnosed as a subtype of TRS (myxoid/round cell liposarcoma, synovial sarcoma, alveolar rhabdomyosarcoma, extraskeletal Ewing sarcoma/primitive neuroectodermal tumor, dermatofibrosarcoma protuberans, low grade fibromyxoid sarcoma, alveolar soft part sarcoma, clear cell sarcoma, angiomatoid fibrous histiocytoma, desmoplastic small round cell tumor, giant cell fibroblastoma, endometrial stromal sarcoma, EMCS, and MCS); unresponsive or intolerable to the standard chemotherapy regimens; receiving no more than four prior chemotherapy regimens; disease progression according to the Response Evaluation Criteria in Solid Tumors (RECIST) version 1.1 confirmed by imaging during the 14 days before the enrollment, compared with the assessment performed during the previous 6 months.

The randomized phase 2 study was approved by the institutional review board at each institution. All participants gave written informed consent before the initiation of the study, which included consent to publish the results of their data. The randomized phase 2 study was conducted in accordance with the ethical principles originating in or derived from the Declaration of Helsinki, International Conference on Harmonization Good Clinical Practice Guidelines, and locally applicable laws and regulations. Trabectedin was supplied by Taiho Pharmaceutical Co., Ltd. (Tokyo, Japan).

### Treatment and assessments

Trabectedin was administered in a standard starting dose of 1.2 mg/m^2^ as a 24-hour continuous intravenous infusion via a central vein on day 1. Each treatment cycle consists of 21 days. The 20-day cycle interval could be extended up to 42 days when adverse events occurred. Dose reduction was allowed in case of grade 3 or 4 adverse events including thrombocytopenia < 25,000/μL, neutropenia < 500/μL with fever and neutropenia < 500/μL persistent for at least 6 days. The study treatment was repeated until disease progression, unmanageable toxicity, subject refusal, or delay for >21 days (one cycle) occurred due to toxicity. In the BSC group, subjects underwent BSC to relieve symptoms and improve QOL; anticancer therapies were prohibited. Tumor assessment by CT or MRI was repeated at weeks 4, 8, 12, 18, and 24, and every 8 weeks thereafter.

Objective response and PFS were assessed according to the RECIST version 1.1 by central radiology imaging review.

The cutoff date for the final data of the randomized phase 2 study was March 2015.

## Results

Between July 11, 2012 and Jan 20, 2014, 76 patients with TRS were enrolled in the randomized phase 2 study, and the full analysis set consisted of 73 subjects. The number of subjects with EMCS and MCS was 2 (2.7 %) and 6 (8.2 %), respectively. Five subjects with EMCS and MCS were allocated to the trabectedin group, and 3 subjects with MCS were allocated to the BSC group. Clinical information of these subjects is presented in Table 1. In the five subjects of the trabectedin group, the median total number of trabectedin cycles was 10.0 (range, 8–22). The median treatment duration from the first administration was 11.7 months (range, 8.9–22.8). Cycle interval of 20 days was extended in all of five subjects, and the major reasons for extension were neutropenia and thrombocytopenia. The median cycle interval was 34.0 days (range, 21–47). In one subject (subject No. 1) the dose of trabectedin was reduced to 1.0 mg/m^2^ in cycle 3 because of adverse event (creatinine phosphokinase increased).

**Table 1:** Clinical information of subjects

Subject No.	Age ranges (years)	PS^a^	Histological type	Primary lesion		Sum of diameter of target lesions (mm)^b^	Time from initial diagnosis to enrolled date (months)	Time to progression in prior systemic chemotherapy (months)
				Site	At baseline			
Trabectedin group								
1	50- < 60	0	EMCS	lower limbs	resected	132.8	94.2	8.7 (IE)
2	60- < 70	1	EMCS	lower limbs	resected	38.4	4.3	1.1 (doxorubicin)
3	<40	0	MCS	neck	conserved	11.3	63.0	NA (neoadjuvant chemotherapy)
4	<40	1	MCS	basal meninges	resected	162.3	82.6	3.7 (doxorubicin)
5	<40	1	MCS	neck	resected	57.3	145.6	NA (neoadjuvant chemotherapy)
Best supportive care group								
6	<40	1	MCS	face	conserved	136.8	116.2	7.0 (doxorubicin and cisplatin)
7	<40	0	MCS	pleura	resected	94.6	2.5	0.7 (VDC)
8	<40	1	MCS	retroperi-toneum	resected	209.3	13.4	NA (neoadjuvant chemotherapy)

*MCS* Mesenchymal chondrosarcoma, *EMCS* Extraskeletal myxoid chondrosarcoma, *PS* performance status, *NA* not applicable, *IE* ifosfamide and etoposide, *VDC* vincristine, doxorubicin and cyclophosphamide

^a^Eastern Cooperative Oncology Group (ECOG) performance status

^b^Assessed by central radiology imaging review

**Fig. 1:**
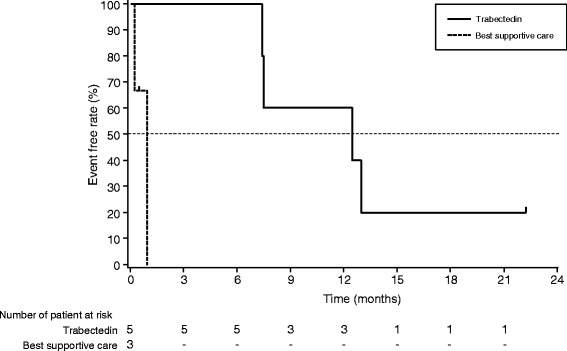
Kaplan-Meier plot of progression-free survival. Progression-free survival of five patients with EMCS and MCS randomized to the trabectedin group (−) and three patients with MCS randomized to the BSC group (−−−)

Median follow-up time of the randomized phase 2 study was 22.7 months, and 1 subject with MCS was still receiving treatment at the final data cutoff. The median PFS of the subjects with EMCS and MCS was 12.5 months (95 % CI: 7.4–not reached) in the trabectedin group, while 1.0 months (95 % CI: 0.3–1.0 months) in MCS subjects of the BSC group (Table 2, Fig. 1). The six-month progression-free rate (PFR) was 100 % in the trabectedin group. The best change in sum of the diameter (%) was ranged from 1 % to −58 %. One subject with MCS (subject No. 3) who received trabectedin treatment for more than 2 years showed partial response (PR). The other subjects in the trabectedin group (two with EMCS and two with MCS) showed stable disease (SD). Progressive disease (PD) was not observed as best response in the trabectedin group. Median overall survival (OS) of EMCS and MCS subjects in the trabectedin group was 26.4 months (range, 10.4–26.4 months), and at the final data cutoff, two subjects were still alive.

**Table 2:** Summary of efficacy

Subject No.	Histological type	Duration of treatment (months)	PFS (months)^a^	Best overall response^a^	Change in sum of the diameter (%)^a, b^	Overall survival (months)	Reason for discontinuation
Trabectedin group							
1	EMCS	15.6	13.0	SD	−1	26.4	progression
2	EMCS	8.9	7.4	SD	−27	10.4	progression
3	MCS	22.8	22.2^*^	PR	−58	23.0^*^	(continued)^c^
4	MCS	10.8	7.5	SD	1	19.2^*^	progression
5	MCS	11.7	12.5	SD	−1	12.5	subject withdrawal^d^
Best supportive care group							
6	MCS	0.5	0.5^*^	NE	−2	24.2	progression
7	MCS	0.3	0.3	PD	12	6.4	progression
8	MCS	1.0	1.0	PD	46	3.4	progression

*MCS* Mesenchymal chondrosarcoma, *EMCS* Extraskeletal myxoid chondrosarcoma, *PFS* progression-free survival, *PR* partial response, *SD* stable disease, *NE* not evaluable, *PD* progressive disease

* Censored observation

^a^Assessed by central radiology imaging review

^b^The best change in sum of the diameter of target lesions from baseline

^c^Participated in another study for continuing trabectedin treatment after termination of the randomized phase 2 study

^d^The subject hoped for different treatment

**Fig. 2:**
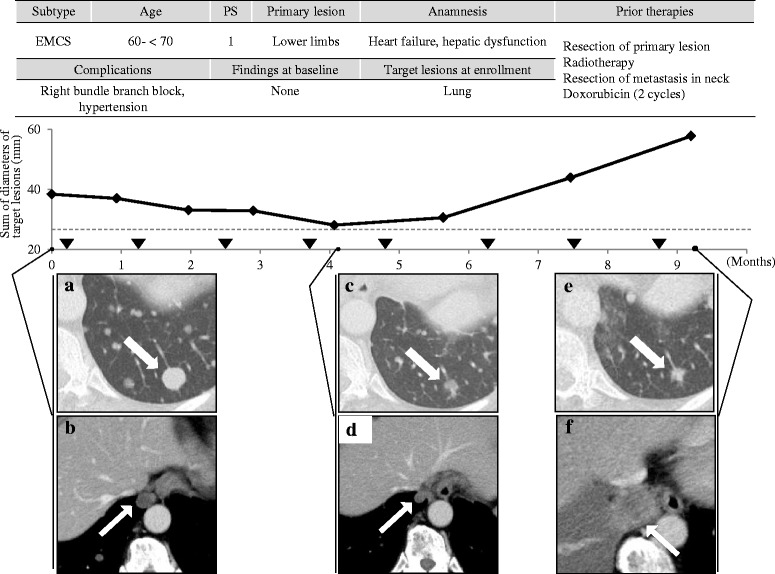
Clinical course of subject No.2. CT images of target lesions in lung at (**a**, **b**) enrollment in the study, (**c**, **d**) 4.0 months after enrollment (27 % decrease in sum of diameters), (**e**, **f**) 9.2 months after enrollment (50 % increase in sum of diameters). EMCS: Extraskeletal myxoid chondrosarcoma. ▼: Administration of trabectedin. ---: Borderline of 30 % decrease in sum of diameters

**Fig. 3:**
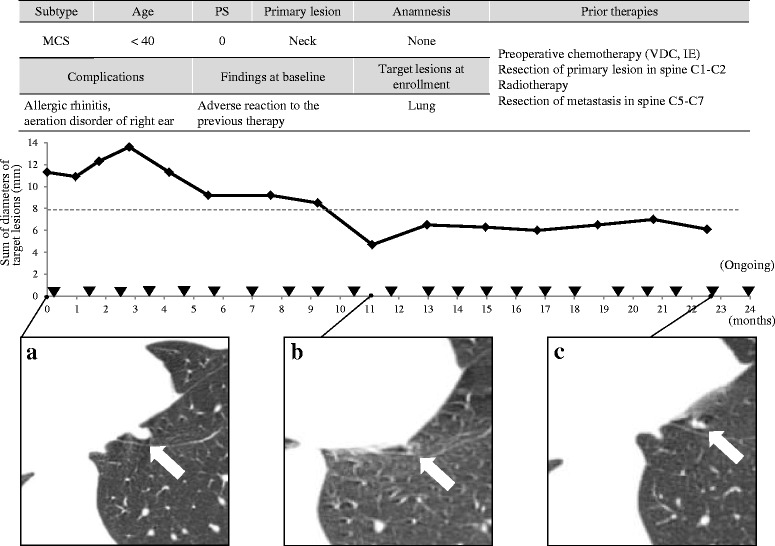
Clinical course of subject No.3. CT images of target lesion in lung at (**a**) enrollment in the study, (**b**) 11.1 months after enrollment (58 % decrease in sum of diameters), (**c**) 22.5 months after enrollment (46 % increase in sum of diameters). MCS: Mesenchymal chondrosarcoma, VDC: vincristine, doxorubicin and cyclophosphamide, IE: ifosfamide and etoposide. ▼: Administration of trabectedin. ---: Borderline of 30 % decrease in sum of diameters

Representative clinical course of both EMCS (subject No. 2) and MCS (subject No. 3) cases with trabectedin treatment are shown in Figs. 2 and 3. Subject No. 2 received eight trabectedin cycles for 8.9 months, showing 27 % shrinkage at 4 months after enrollment. Subject No. 3 had a target lesion of 11 mm in lung at baseline which had increased until the subject started trabectedin treatment. Subject No. 3 received 22 trabectedin cycles over 22.8 months, showing increasing tumor size during first 3 months, and then shrinking to 58 % at 11.0 months after enrollment. Of another three subjects, two showed the best shrinkage within first 2 months, and their lesions thereafter gradually grew. The worst change in the diameter of their lesions was ranged from 9.2 to 39.5 %.

No subjects withdrew from the study due to toxicity, and no deaths were assessed as drug-related. Adverse drug reactions in the trabectedin group are shown in Table 3.

**Table 3:** Adverse drug reactions

		*N* = 5	
		≥G1	≥G3
		*n* (%)	*n* (%)
Any adverse drug reactions		5 (100.0)	5 (100.0)
Clinical findings			
	Nausea	4 (80.0)	2 (40.0)
	Malaise	4 (80.0)	0 (0.0)
	Vomiting	3 (60.0)	0 (0.0)
	Decreased appetite	3 (60.0)	0 (0.0)
	Constipation	3 (60.0)	0 (0.0)
	Oedema peripheral	3 (60.0)	0 (0.0)
	Anaemia	2 (40.0)	2 (40.0)
	Oral discomfort	2 (40.0)	0 (0.0)
	Stomatitis	2 (40.0)	0 (0.0)
	Pyrexia	2 (40.0)	0 (0.0)
	Dysgeusia	2 (40.0)	0 (0.0)
	Headache	2 (40.0)	0 (0.0)
Abnormal laboratory values			
	Neutrophil count decreased	5 (100.0)	5 (100.0)
	Platelet count decreased	4 (80.0)	1 (20.0)
	White blood cell count decreased	4 (80.0)	4 (80.0)
	Alanine aminotransferase increased	2 (40.0)	2 (40.0)

Adverse drug reactions occurring in ≥ 2 subjects are shown

Grade was assessed according to Common Terminology Criteria for Adverse Events (CTCAE) version 4.03

## Discussion

EMCS is a rare soft tissue sarcoma that accounts for less than 3 % of all soft tissue sarcomas, which was first reported by Enzinger in 1972. EMCS contains an abundant mucinous stroma in which malignant chondroblast-like tumor cells grow in a lobulated manner. No clear differentiation by the tumor cells into cartilage is seen, and histologically EMCS is classified as soft tissue sarcoma with no clear differentiation tendencies. A high rate of characteristic chromosome translocations, i.e., t(9;22)(q22;q12) and t(9;17)(q22;q11), and resulting fused genes, i.e., *EWSR1-NR4A3* and *TAF15-NR4A3*, are seen in the tumors. EMCS usually grows slowly, but the oncologic properties are often unclear. The basic treatment of EMCS is wide resection, and the benefit of chemotherapy or radiotherapy has never been established. A report by Ogura et al. [11] stated that ifosfamide-containing chemotherapy was performed in four high-grade EMCS cases, and their responses were not available (NA) in one case, SD in one case, and PD in two cases. The efficacy of the chemotherapy was reported as inadequate.

MCS, on the other hand, is a subtype of chondrosarcoma that consists of proliferation of poorly differentiated small round cells and well differentiated cartilage tissue. In recent years, a tumor-specific fusion gene *HEY1-NCOA2* that occurs as a result of a chromosome translocation was identified by Wang et al. [9]. The largest proportion of MCS, over 70 %, arises from bone, and less than 30 % originate in soft tissue. However, MCS originated in soft tissue is said to be more than reported, in the following respects. First, it is sometimes difficult to determine whether the origin of an MCS of the spine, etc., is bone or dura mater. Second, whereas many MCSs are of dural origin, some dural origin MCS may have been included among those of bony origin. Not surprisingly, the treatment of first choice for MCS is wide resection. Although occasional cases have shown disease control by chemotherapy and/or radiotherapy, no consensus about use of chemotherapy or radiotherapy has been reached to date.

In the randomized phase 2 study on which the present analysis was based, trabectedin was introduced into five subjects with advanced EMCS and MCS. All of five subjects showed improved disease control, in contrast with the three subjects with MCS in the BSC group. It should be noted that long-term disease control was observed in the all subjects with EMCS and MCS in the trabectedin group. Moreover, the PFS of EMCS and MCS in the trabectedin group seemed to be better than that of myxoid liposarcoma, which has been demonstrated to be highly responsive to trabectedin [5, 12]. Le Cesne et al. [13] reported that median PFS and OS of chondrosarcoma were 6.267 months (95 % CI: 0.000–15.935) and 21.400 months (95 % CI: 9.641–33.159), which seem to be slightly longer than those of liposarcoma [median PFS; 6.067 months (95 % CI: 4.488–7.645), median OS; 15.000 months (95 % CI: 11.033–18.967)]. Our data in EMCS and MCS showed similar PFS and OS.

We observed anti-tumor effect (PR or SD) in all subjects in the trabectedin group. One subject with MCS showed promising response, with tumors shrinking more than 50 %.

Le Cesne et al. [5] retrospectively investigated the efficacy of trabectedin in 81 subjects with TRS (synovial sarcoma, myxoid-round cell liposarcoma, alveolar soft part sarcoma, endometrial stromal sarcoma, and clear cell sarcoma, not including EMCS and MCS). The results showed median PFS was 4.1 months (95 % CI: 2.8–6.1), the six-month PFR was 40 %, and the OS of TRS was 17.4 months (95 % CI: 11.1–23.2). The present analysis seems to show better results.

In research in vitro, trabectedin has been reported to inhibit the transcription factor function of fused proteins produced as a result of the chromosome translocations in some human bone and soft tissue sarcoma cell lines that have a chromosome translocation [2, 3]. This appears to be one of the mechanisms by which trabectedin exhibits a strong antitumor effect against soft tissue sarcomas that have chromosome translocations.

There are limited data about the efficacy of chemotherapy in patients with EMCS or MCS, because EMCS and MCS are very rare tumors, and no consensus has been reached in regard to the efficacy of existing chemotherapy for either of these tumors. Additionally, the starting trabectedin dose of 1.2 mg/m^2^ used in the present analysis of the phase 2 study was based on the phase 1 study [14], which is lower than the approved initial dose of 1.5 mg/m^2^, and corresponds to the approved first reduction dose in case of toxicity for the treatment of advanced STS in the European Union. Limitations of our results include the small sample size and resultant difficulty to generalize. Our findings suggest that it is necessary to evaluate efficacy of trabectedin for more patients with EMCS and MCS.

## Conclusions

In conclusion, this sub-analysis shows that trabectedin is effective for patients with EMCS and MCS compared with BSC. The efficacy results were better than previously reported data of TRS. In the study on which this analysis was based, trabectedin was introduced into subjects with advanced EMCS and MCS, and showed long-term disease control in all the subjects. Tumor shrinking effects were also observed, and one subject who showed PR has undergone long-term treatment. These facts suggest that trabectedin become an important choice of treatment for patients with advanced EMCS and MCS who failed or intolerable to standard chemotherapy.

## Abbreviations

BSC, best supportive care; EMCS, extraskeletal myxoid chondrosarcoma; MCS, mesenchymal chondrosarcoma; NA, not available; OS, overall survival; PD, progressive disease; PFR, progression-free rate; PFS, progression-free survival; PR, partial response; RECIST, Response Evaluation Criteria in Solid Tumors; SD, stable disease; TRS, translocation-related sarcomas

## References

[1] D’Incalci M, Badri N, Galmarini CM, Allavena P (2014). Trabectedin, a drug acting on both cancer cells and the tumour microenvironment. Br J Cancer.

[2] Forni C, Minuzzo M, Virdis E, Tamborini E, Simone M, Tavecchio M (2009). Trabectedin (ET-743) promotes differentiation in myxoid liposarcoma tumors. Mol Cancer Ther.

[3] Grohar PJ, Griffin LB, Yeung C, Chen QR, Pommier Y, Khanna C (2011). Ecteinascidin 743 interferes with the activity of EWS-FLI1 in Ewing sarcoma cells. Neoplasia.

[4] Xia SJ, Barr FG (2005). Chromosome translocations in sarcomas and the emergence of oncogenic transcription factors. Euro J Cancer.

[5] Le Cesne A, Cresta S, Maki RG, Blay JY, Verweij J, Poveda A (2012). A retrospective analysis of antitumour activity with trabectedin in translocation-related sarcomas. Eur J Cancer.

[6] Kawai A, Araki N, Sugiura H, Ueda T, Yonemoto T, Takahashi M (2015). Trabectedin monotherapy after standard chemotherapy versus best supportive care in patients with advanced, translocation-related sarcoma: a randomised, open-label, phase 2 study. Lancet Oncol.

[7] de Alava E (2007). Molecular pathology in sarcomas. Clin Transl Oncol.

[8] Hisaoka M, Hashimoto H (2005). Extraskeletal myxoid chondrosarcoma: updated clinicopathological and molecular genetic characteristics. Pathol Int.

[9] Wang L, Motoi T, Khanin R, Olshen A, Mertens F, Bridge J (2012). Identification of a novel, recurrent HEY1-NCOA2 fusion in mesenchymal chondrosarcoma based on a genome-wide screen of exon-level expression data. Genes Chromosomes Cancer.

[10] Morioka H, Weissbach L, Vogel T, Nielsen GP, Faircloth GT, Shao L (2003). Antiangiogenesis treatment combined with chemotherapy produces chondrosarcoma necrosis. Clin Cancer Res.

[11] Ogura K, Fujiwara T, Beppu Y, Chuman H, Yoshida A, Kawano H (2012). Extraskeletal myxoid chondrosarcoma: a review of 23 patients treated at a single referral center with long-term follow-up. Arch Orthop Trauma Surg.

[12] Grosso F, Jones RL, Demetri GD, Judson IR, Blay JY, Le Cesne A (2007). Efficacy of trabectedin (ecteinascidin-743) in advanced pretreated myxoid liposarcomas: a retrospective study. Lancet Oncol.

[13] Le Cesne A, Ray-Coquard I, Duffaud F (2015). for French Sarcoma Group. Trabectedin in patients with advanced soft tissue sarcoma: a retrospective national analysis of the French Sarcoma Group. Eur J Cancer.

[14] Ueda T, Kakunaga S, Ando M (2014). Phase I and pharmacokinetic study of trabectedin, a DNA minor groove binder, administered as a 24-h continuous infusion in Japanese patients with soft tissue sarcoma. Invest New Drugs.

